# Whole-Genome Analysis of De Novo Somatic Point Mutations Reveals Novel Mutational Biomarkers in Pancreatic Cancer

**DOI:** 10.3390/cancers13174376

**Published:** 2021-08-30

**Authors:** Amin Ghareyazi, Amir Mohseni, Hamed Dashti, Amin Beheshti, Abdollah Dehzangi, Hamid R. Rabiee, Hamid Alinejad-Rokny

**Affiliations:** 1 Bioinformatics and Computational Biology Laboratory, Sharif University of Technology, Tehran 11365, Iran; amin.ghareyazi@sharif.edu (A.G.); amir.mohseni@sharif.edu (A.M.); dashtih@ce.sharif.edu (H.D.); 2 Department of Computing, Macquarie University, Sydney, NSW 2109, Australia; amin.beheshti@mq.edu.au; 3 Department of Computer Science, Rutgers University, Camden, NJ 08102, USA; i.dehzangi@rutgers.edu; 4 Center for Computational and Integrative Biology, Rutgers University, Camden, NJ 08102, USA; 5 BioMedical Machine Learning Lab (BML), The Graduate School of Biomedical Engineering, The University of New South Wales, Sydney, NSW 2052, Australia; 6 UNSW Data Science Hub, The University of New South Wales, Sydney, NSW 2052, Australia; 7 Health Data Analytics Program, AI-Enabled Processes (AIP) Research Centre, Macquarie University, Sydney, NSW 2109, Australia

**Keywords:** pancreatic cancer, cancer subtype identification, somatic point mutations, genotype and phenotype characterization, therapeutic targets, personalized medicine

## Abstract

**Simple Summary:**

Many studies have identified cancer subtypes based on the cancer driver genes, or the proportion of mutational processes in cancer genomes, however, none of these cancer subtyping methods consider these features together to identify cancer subtypes. Accurate classification of cancer individuals with similar mutational profiles may help clinicians to identify individuals who could receive the same types of treatment. Here, we develop a new statistical pipeline and use a novel concept, “gene-motif”, to identify five pancreatic cancer subtypes, in which for most of them, targeted treatment options are currently available. More importantly, for the first time we provide a system-wide analysis of the enrichment of de novo mutations in a specific motif context of the driver genes in pancreatic cancer. By knowing the genes and motif associated with the mutations, a personalized treatment can be developed that considers the specific nucleotide sequence context of mutations within responsible genes.

**Abstract:**

It is now known that at least 10% of samples with pancreatic cancers (PC) contain a causative mutation in the known susceptibility genes, suggesting the importance of identifying cancer-associated genes that carry the causative mutations in high-risk individuals for early detection of PC. In this study, we develop a statistical pipeline using a new concept, called gene-motif, that utilizes both mutated genes and mutational processes to identify 4211 3-nucleotide PC-associated gene-motifs within 203 significantly mutated genes in PC. Using these gene-motifs as distinguishable features for pancreatic cancer subtyping results in identifying five PC subtypes with distinguishable phenotypes and genotypes. Our comprehensive biological characterization reveals that these PC subtypes are associated with different molecular mechanisms including unique cancer related signaling pathways, in which for most of the subtypes targeted treatment options are currently available. Some of the pathways we identified in all five PC subtypes, including cell cycle and the Axon guidance pathway are frequently seen and mutated in cancer. We also identified Protein kinase C, EGFR (epidermal growth factor receptor) signaling pathway and P53 signaling pathways as potential targets for treatment of the PC subtypes. Altogether, our results uncover the importance of considering both the mutation type and mutated genes in the identification of cancer subtypes and biomarkers.

## 1. Introduction

Pancreatic cancer (PC) is the third leading cause of death among all cancers, with the lowest survival rate of 9% [1]. PC is predicted to become the second leading fatal cancer [2]. Moreover, the advancement achieved in increasing survival time for lung and pancreatic cancers has been slow compared to other types of cancers [1]. PC can be categorized into different subtypes based on specifications of mutations, molecular profile, and histopathological characteristics. Such subtypes can have different mechanisms and different responses to treatments [3]. Therefore, identifying subtypes can lead to the identification of unique biomarkers, more effective treatment approaches, and also directly contributing to personalized medicine. Identification of subtypes for breast [4] and lung [5] cancers has led to finding new effective treatments, and better-targeted drugs. Moreover, determining subtypes can potentially play a vital role in increasing prognostic accuracy for pancreatic cancer.

During the last decade, a wide range of studies has been conducted to identify corresponding pancreatic cancer subtypes with a special focus on gene expression profiles as features [6]. In 2011, Collisson et al. proposed a combined analysis to tackle the limitations of the number of tumor samples for PC subtype identification [7]. They used combined analysis of transcriptional profiles of primary pancreatic ductal adenocarcinoma (PDAC is an exocrine type of pancreatic cancer) from several studies, along with the human and mouse PDAC cell lines. By using gene expression, they identified three subtypes and 62-gene signatures for PC [7]. In 2015, Moffit et al. expanded the Collison et al. work by adding stromal classifications [8]. They also employed the global gene expression analysis with RNA sequencing validation and proposed two subtypes for each stroma-specific and tumor-specific group. Remarkably, they reported an overlap between one of their identified tumor-specific subtypes called “classical” and the Collisson et al. classical subtype [8]. Both of these studies were served as the basic foundation of the Bailey et al. research [9]. They proposed an integrated genomic analysis by using deep-exome and whole-genome with gene copy number analysis, along with RNA-seq validation. They identified four subtypes, namely, squamous, pancreatic progenitor, immunogenic, and Aberrantly Differentiated Endocrine Exocrine (ADEX) for pancreatic cancer. Furthermore, they specified several gene-based categories according to similarities among their pathways [9]. In another study, Sivakumar et al. used expression profiles of 204 ICGC and 149 TCGA samples to tackle this problem [10]. Using a network-based and community detection method, they identify three main subtypes for PC. In their study, the focus was the activity and characteristics of the *KRAS* gene in PC. In one of the latest works on PC subtyping, Puelo et al. used gene expression of 309 resected primary PDAC and identified five different subtypes based on features of cancer cells and the tumor microenvironment [11].

A mentioned earlier, pancreatic subtype identification by using the gene expression data, is widely popular. However, gene expression is tissue and time specific. It means that the gene expression of tissue can vary at different time points. Moreover, gene expressions of different tissues are different at a single time point [12]. Hence, relying on gene expression for cancer subtype identification might not provide a general and reliable result. On the other hand, somatic mutations, as important players in cancer development and disease progression, are less affected by factors that can influence gene expression [13].

Recently, Kuijjer et al. used somatic point mutations for identifying mutational diversities in pan-cancer to find new types of cancer among all cancers [14]. They classified patients with similar mutation profiles into subgroups by applying biological pathways [14]. In another pan-cancer study, Kuipers et al. proposed a method for finding subgroups of cancer based on interactions of mutations [15]. In the field of pancreatic cancer subtype identification, Waddell et al. provided a pipeline for analysis of the pattern of structural variations (including copy number variations, somatic and germline mutations) in 100 PDAC samples [16]. They identified four main subtypes and named them as “stable”, “locally rearranged”, “scattered” and “unstable”. They have not included any samples from the exocrine type (a rare type of PC) in their study.

**Figure 1:**
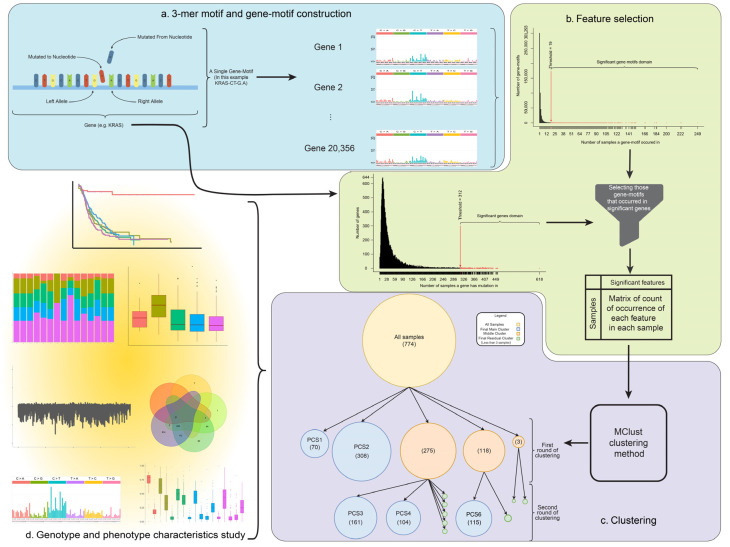
The workflow of pancreatic cancer subtype identification and clustering tree. In the top left, an overall view of the 3-mer motif and the gene-motif concept is illustrated. (**a**) At first, we construct features named gene-motifs based on the 3-mer motif and the gene that motif has occurred in. These features were constructed for all samples and in all of their protein-coding genes. In the top right, the feature selection process is illustrated. (**b**) We calculated the number of samples each gene-motif has occurred in, and based on their distributions, we found the most frequent (and hence significant) gene-motifs. We also found the most frequent mutated genes or significantly mutated genes to filter out those gene-motifs that have not occurred in significant genes. This leads to significant features for clustering. (**c**) The clustering process and tree is illustrated. After constructing a matrix of occurrence for each feature in each sample, (each cell indicates whether a feature has occurred in a sample or not) the Mclust algorithm was employed to cluster samples into subtypes. After two rounds of clustering, five main subtypes revealed themselves. (**d**) Finally, comprehensive genotype and phenotype characteristic study was performed to find differences and/or commonality in subtypes (bottom left). This includes gene association, mutational signature, deep mutational profile investigation, finding DEGs, survival analysis, etc.

In 2013, Alexandrov et al. published a paper and showed that there are 78 mutational signatures in cancers, most of them associated with a specific molecular mechanism to uncover the causality behind somatic point mutations across the genome [17]. The proposed concept provided the importance of motifs in the analysis of somatic point mutations in cancer genomics. To the best of our knowledge, nobody has used the context of mutations in highly mutated genes for cancer subtype identification. As we discussed above, multiple groups identified 3–5 PC subtypes, however, they did not consider the underlying mutational context to cluster affected patients. In this study, we perform an integrative analysis using “gene-motif” information extracted from somatic mutations to tackle this problem. We hypothesize that accurate PC subtypes identification depends on both mutations and their corresponding motifs as well as the respective mutated genes. Therefore, we proposed a feature called “gene-motif” to accurately identify subtypes in pancreatic cancer. We conducted our integrative analysis on the dataset from ICGC consortia consisting of 774 samples with PC. This dataset is by far larger than those used in the previous studies which demonstrate the comprehensiveness of this study, and generality of our findings. To build our model, we first identified candidate gene-motifs as our features to cluster the PC samples. Such features were selected based on the empirical distribution of the number of mutations in gene-motifs. After the candidate gene-motifs were identified, we used a model-based clustering approach for clustering the PC samples to identify the subtypes. We identified five subtypes with distinguishable relations between candidate genes, phenotype, and genotype characteristics of PC subtypes. We also identified subtype-specific mutational signatures and compared them with the latest COSMIC [18] mutational signatures to investigate the molecular mechanisms behind mutations in each subtype. We also investigated the mutational load in coding genes to identify subtype-specific genes. Our gene ontology and pathway analyses also demonstrate common and subtype-specific terms. We next analyzed RNA-Seq gene expression data of PC samples and investigated the difference of gene expression between the identified subtypes. We also conducted a complete survival analysis and studied the effects of histopathological information on survival time prediction. An overview of the analysis pipeline used in this study is demonstrated in Figure 1. Our proposed model and its related codes are publicly available online at Github [19] https://github.com/bcb-sut/Pancreatic-Cancer-Subtype-Identification, accessed on 6 January 2021.

## 2. Materials and Methods

### 2.1. Data

Simple somatic mutation data for all pancreatic cancer projects from ICGC [20]. This dataset includes information of 17,284,164 simple somatic mutations of 827 samples. RNA-seq gene expression data of 534 PC samples were also available from the ICGC data.

### 2.2. Data Cleaning and Filtration

We collected all the information about the position of simple somatic mutation (SSM) from the ICGC dataset which includes chromosomal position, start and end position, gene ID, transcript ID, reference genome allele, and mutated allele. We also extracted the information such as consequence type and project code from ICGC for analysis. Only simple somatic point mutations were considered for further analyses.

To find the 3-nucleotide motifs, we used BSgenome.Hsapiens.UCSC.hg19, GenomicRnages [21], and SomaticSignatures [22] packages in the R programming language. 3-nucleotide motifs and their respective genes were concatenated as the clustering features. For the analysis of gene expression data, samples that had the same sample ID from five subtypes were used. Finally, the RNA-seq data from 307 samples remained for the downstream analysis. Consequently, PCS1 contains 22 samples and PCS2, PCS3, PCS4 and PCS5 contains 10, 108, 76, 91 samples, respectively. Some genes in this dataset had more than one value for some samples, and we used the mean value from them. Protein coding genes that had NA values were removed from the analysis.

### 2.3. Feature Selection

We used gene-motifs [23] as the feature for our analyses. For each gene, we counted the number of mutated samples. Moreover, for each gene-motif, we counted the number of samples in which that gene-motif has occurred. Afterward, we used the empirical distribution of these counts to assign the probability of occurrence to each gene and gene-motif. In this step, genes and gene-motifs with probability <= 0.01 were considered to be significant genes and gene-motifs. For our dataset, the genes with mutations in at least 312 samples, constitute about 0.01 of all protein-coding genes. Additionally, those gene motifs that have occurred in at least 19 samples, are about 0.01 of all possible gene-motifs. As a result, 203 genes and 5704 gene-motifs were above their respective criteria and considered to be significant gene and gene-motif, respectively. All the significant gene and gene-motifs are provided in Tables S1 and S2. Finally, the significant gene-motifs that their respective genes were members of the significant gene set, were selected as the clustering features. A matrix consisting of all the 4211 candidate gene-motifs (Table S3) for all samples, was used for clustering.

### 2.4. Clustering Method

In this study, we used the Mclust method [24] to cluster samples. To identify the optimum number of clusters, Mclust utilizes a Gaussian mixture model with the Bayesian Information Criterion (BIC) that is the best criterion for finding optimal number of clusters in mixture models [25,26]. We used the implementation of Mclust in the CRAN package. Mclust does not make any assumption on the parameters of distribution function for features, and it does not make any assumption on the number of clusters. These properties make it a suitable clustering method. Mclust was applied recursively until no meaningful new cluster was generated. A cluster is assumed to be meaningful if it contains at least 1% of the total number of samples (at least 7 samples). Hence, clusters with less than this threshold were outliers. To illustrate the segregation and differentiation between clusters we performed a PCA analysis. The first two principal components of our data demonstrate that clusters are separated well (Figure S1).

The results of our clustering method are shown in Figure 1. On the first round of clustering, 5 clusters with sizes of 70, 308, 275, 118, and 3 samples were found. On the second round of clustering, clusters with 70 and 308 samples did not break into smaller clusters. Hence, these two were considered as the main subtypes and were named PCS1 and PCS2. On the other hand, the cluster with 275 samples, split into 2 clusters with 161 (called PCS3) and 104 (called PCS4) samples, and 5 other clusters with 2 samples. The cluster with 118 samples that were found in the first round was divided into 2 clusters with 115 (called PCSS5) and 3 samples. Other small clusters with less than 7 samples were considered as outliers.

### 2.5. Differential Analysis

We used the differential analysis to investigate differences in rates of samples with mutation, in the protein-coding gene. We counted the number of samples with a mutation in each gene, for all 5 subtypes. The rates of each gene in each cluster were deducted from its rate in other subtypes. The same process was performed on clustering features.

### 2.6. Mutational Signature Analysis

We used the CANCERSIGN package in R [27] to calculate the mutational signatures of pancreatic cancer, and the level of exposures of each sample to each signature. This tool implements the Non-negative Matrix Factorization (NMF) method to find patterns of 3-nucleotide motifs among samples. The signatures were extracted for all pancreatic cancer samples as well as each subtype, individually. The input to CANCERSIGN is a matrix of samples in rows, and features (including chromosome, mutation position, reference allele, and mutated to allele) in columns. The analysis was performed with the number of signatures ranging from 1 to 15, and the maximum bootstrap iterations for each step was set to 780. The cosine distance was used to compare the signatures. The evaluation plot of deciphering 3-mer mutational signatures is provided in Figure S2. 

### 2.7. Motif Analysis 

Each mutation and its context (left and right alleles of a mutated position), and the substituted nucleic acid in that position, constructs a 3-nucleotide motif. There are 96 combinations of 3-nucleotide motifs. Patterns of these 3-nucleotide motifs can provide important biological information about the molecular mechanism [28,29,30]. The relative frequency of motifs was calculated cumulatively for subtypes (Figure S3), and common associated genes (Table S4). Motif rates of outlier clusters are provided in Figure S4. Tests for the piqued motifs in common associated genes were undertaken by utilizing the Fisher exact test. For example, we counted the number of samples that had the motif TA-A.A and also were in PCS1 (or PCS3) for the gene NRG1. We tested the relationship between these two dichotomous variables by Fisher’s exact test.

### 2.8. Transcript Type Analysis

Each mutation can affect one or more transcripts of the gene. The differences in subtypes indicate different effects on the organisms. To investigate these rates, the relative frequency of samples in each subtype with a mutation in each transcript, for all protein-coding genes were calculated.

### 2.9. Gene Association

The association of protein-coding genes to each subtype was done by utilizing Fisher’s exact test. This test was applied to identify mutated genes as the potential biomarker for each subtype. To identify such association using Fisher’s exact test we used a 2 × 2 contingency matrix. This matrix contains information relating to the number of samples in all possible combinations of two variables. These variables are (1) being categorized as a member of a certain subtype or not, and (2) having at least one mutation in a given gene or not. This test was used for all genes in all subtypes. To find a significant threshold for *p*-values, a permutation test was conducted. To do this, first, a table of the number of mutated samples for each gene was randomly generated, such that their total number over all the genes remains the same. This table was created for all subtypes. Second, Fisher’s exact test was conducted as described above on all genes and for all subtypes. Third, these steps were repeated 10,000 times. Fourth, for each gene, 10,000 *p*-values were generated. We considered the *p*-value of the lowest 0.05 percent of these numbers as the significance threshold. For the final step, we chose the genes that were mutated at least in 50% of samples of their respective subtype and considered them as associated genes to subtypes (Table S5). A Venn diagram of common associated genes in subtypes is provided in Figure S5.

### 2.10. Gene Expression Analysis

Raw read count of 19,104 protein-coding genes from 307 samples was gathered in a matrix. The DESeq2 package and its guideline were used for finding differentially expressed genes (DEGs) between the groups [31]. Genes with a *p*-value of less than 0.05 were considered as significantly differentially expressed genes. First, significant DEGs of PCS1 were compared to all other subtypes. This was also done for other subtypes. Second, in five sets of DEGs, unique genes and common genes were distinguished, as shown in the Venn diagram of Figure S6. Those genes that are only in the respective set of each subtype, are considered as uniquely differentially expressed genes (UDEGs).

### 2.11. Gene Ontology and Pathway

Gene ontology and pathway analyses were performed by using the Enrichr online tool (https://amp.pharm.mssm.edu/Enrichr/ (accessed on 6 January 2020)) [32]. Associated genes to each subtype were used as input to this tool. For the *p*-value adjustment, the Benjamini–Hochberg method was employed. Only ontologies with FDR < 0.05 were considered.

### 2.12. Gender and Project Code Analysis

Project codes of the ICGC database contain information related to the types of pancreatic cancer and the region where the data is gathered. We can also retrieve the gender of donors in the meta-data of donors in this database. Here, this information was used to investigate the possible relation between subtypes, their living location, and their gender. We used genders and project codes in each subtype. Our samples were either male or female, and belong to 4 project codes, namely Pancreatic Cancer Ductal adenocarcinoma from Australia (PACA-AU), Pancreatic Cancer from Canada (PACA-CA), Pancreatic Cancer Endocrine neoplasms from Australia (PAEN-AU), and Pancreatic Endocrine neoplasms from Italy (PAEN-IT). Frequencies of genders and project codes are provided in Tables S6 and S7. We used the frequency of samples of each project in each subtype to investigate if any meaningful relationship between this information and subtypes can be observed.

### 2.13. Literature Search for Non-Coding Interacting Genes

Our literature searches to identify cancer-associated genes were focused on human studies and English language publications available in the PubMed, Scopus, and Web of Science. We also used data and text mining techniques to extract additional related studies [18,33,34,35,36,37,38]. A decision tree approach and a knowledge-based filtering system technique were also used to categorize the texts from the literature search [37,39]. The search terms included “noncoding RNA” or “lncRNAs” or “genes name + cancer”. “BC” or “breast carcinoma” and “breast neoplasm”.

### 2.14. Survival Analysis

We used the donor survival time as the overall survival time for each donor, and vital status was used for the Kaplan–Maier method to estimate the overall survival of each subtype. To conduct survival analysis, we discarded the data for donors that had missing or NA values for survival time (or survival time with zero days) or vital status.

Here, we also report the mean and median of overall survival for each subtype and their 95% confidence interval in Table S8. We also studied differences of overall survival between subtypes by using the log-rank test, Breslow test [40], and Taron-Ware test [41]. Pairs of subtypes with a *p*-value of less than 0.05 were considered to be unequal in terms of their survival curves. Results are provided in Table S9.

We also used the Cox proportional hazards model to evaluate the prognosis power of subtype indicators for survival prediction [15]. We applied several models with adjustment for age at diagnosis, tumor stage, tumor grades, and subtype indicator variables to survival data. As a result, tumor stage categories were aggregated into 5 categories (stages I to IV and stage X; indicating samples with unknown staging status). We also assembled grade categories in 7 levels, namely, (1) well differentiated, (2) moderately differentiated, (3) poorly differentiated, (4) undifferentiated, (5) NET well differentiated, (6) NET moderately differentiated, and (7) NET poorly differentiated. Samples without information or false values (e.g., 0 or NA) were removed, leaving 625 samples for analysis.

A full model with adjustment for all variables is presented in Table S10. We also report the *p*-values of the coefficient and likelihood ratio test of comparison with the null model (a model without predictor) for each case. We also tested the effect of each variable on survival prediction in the complete model using the likelihood ratio test. A likelihood ratio test was conducted to compare the reduced model and complete model to assess the effect of the removed variable in the complete model. To do this, variables were removed from the complete model, one at a time, to generate the reduced model. A likelihood ratio test comparing the complete model and the reduced model was employed to evaluate the effect of the removed variables on the survival time. *p*-values of these tests are reported in Table S11.

### 2.15. Comparing the Overall Mutation Rate in Subtypes

We conducted an independent *t*-test to explore the statistical differences between subtypes, in terms of the relative frequency of samples with mutations in significant genes, and all protein-coding genes. We also investigated the rate of mutation in significant gene-motifs, and significant features (Table S12). After calculating the rate of samples that had a mutation in each protein-coding gene for all subtypes, these rates were tested in pairs, to compare means of mutation rates in subtypes. The same process was also carried out for significant genes, significant gene-motifs, and significant features.

## 3. Results

### 3.1. Background Model to Identify Subtypes in Pancreatic Cancer

In this study, we used somatic point mutations in pancreatic cancer patients from the ICGC dataset. This dataset contains information on mutations of PC samples, based on the whole genome sequencing technology. These samples are collected from three different regions including Australia, Canada, and Italy. In total, 57% of these samples were collected from male and 43% from female donors. After the data cleaning process, (see methods section) information of 774 samples was selected for further analysis.

Since somatic mutations are one of the important factors in the progression and development of cancers [13], they were the main point of attention for integrative analysis of PC subtype identification. There are different mutational processes in living organisms causing mutations across the genome. These molecular mechanisms may cause mutations based on the adjacent bases of a locus [42]. This means that mutational mechanisms may act differently based on neighboring positions of a locus. According to [28], there are 96 possible mutation types in 3-nucleotide motifs (the original nucleic acid, the nucleic acid which it has mutated to, and its right and left alleles). Rates of each 3-nucleotide motif may vary in genes. To count each 3-nucleotide motif in each gene separately, we constructed a new feature called “gene-motif”. For example, KRAS-CT-A.G refers to the number of CT-A.G 3-nucleotide motif in the *KRAS* gene. “CT-G.A” stands for a mutation point that its reference nucleobase is C, mutated to T, its right nucleobase is A, and its left nucleobase is G. The main reason for studying this type of variation is that by depending solely on genes we will overlook the information stored on mutations themselves. An overview of the gene-motif context is shown in Figure 1.

In the first step, we used empirical distributions to select 4211 significant gene-motifs as the features for clustering (see methods section). A full list of clustering features is provided in Table S3. We then adopted the *Mclust* algorithm [24] (see methods section) and identified five main clusters in pancreatic cancer individuals, which we refer to as PCS1, PCS2, PCS3, PCS4, and PCS5. A PCA analysis also confirmed that clusters are well identified and separated (Figure S1). The clustering tree for the process of finding these subtypes is available in Figure 1. As Figure 1 shows, there are 70, 308, 161, 104, and 115 samples for subtypes PCS1, PCS2, PCS3, PCS4, and PCS5, respectively.

In the following sections, genotype and phenotype characteristics of these five PC subtypes are analyzed and discussed in detail to represent their unique and differentiative properties.

### 3.2. Relative Frequency of Mutations in the PC Subtypes 

**Figure 2:**
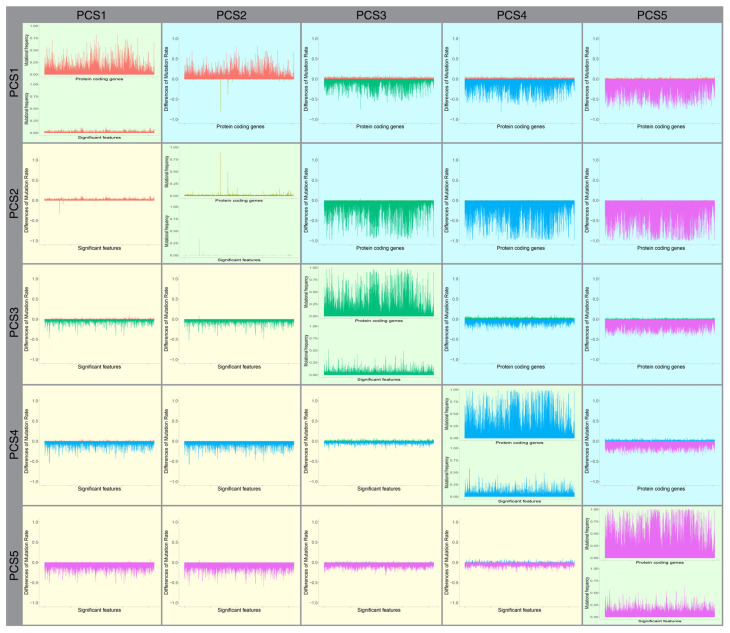
The mutation rate in subtypes and their differences. Bar plots in green tiles exhibit mutation frequency in protein-coding genes and significant features. Yellow tiles include bar plots of differences of mutation rate for significant features and blue tiles include differences in mutation rate in protein-coding genes. For example, the bar plot in the tile which is in PCS2 column and PCS2 row represents the difference of mutation rate in protein-coding genes in PCS2 and PCS1. The color of bars in differential bar plots represents the subtype with the higher mutation rate. For instance, if a bar is differential, the bar plot is red, and PCS1 has the higher mutation rate in that comparison (the same color as bars in the bar plot of mutation rate, in that subtype).

We investigated the differences in the mutational rate of all protein-coding genes and also significantly mutated genes to determine the mutational specification of our identified subtypes and explore if the PC subtypes are different concerning their mutational load. As shown in Figure 2, the level of mutational load in all coding genes and significantly mutated genes within the identified subtypes is different. This difference is much clearer in subtypes PCS1, PCS2, and PCS3, while PCS4 and PCS5 have almost similar patterns. To investigate the mutational difference between the subtypes, we performed a differential mutation analysis (see method section) to explore the difference between the frequency of mutations in each gene and gene-motifs in each pair of subtypes. As shown in Figure 2, the difference between PC samples in subtypes PCS4 and PCS5 is more significant, indicating they are correctly grouped in two different subtypes. The figure also shows that the mutational frequency of the candidate gene-motifs in the subtypes are different, suggesting the different mutational mechanisms among the identified subtypes. We also conducted an independent sample *t*-test and determined when the difference between subtypes in the differential mutation analysis is more evident (see method section). We performed this analysis in three different scenarios by using all protein-coding genes, significant genes, and significant gene-motifs. Our results demonstrate that all subtypes are significantly different in terms of their mutational load in protein-coding genes and the significant gene-motifs (Table S12).

### 3.3. Biological Characterization of Each Subtype

We next investigated different aspects of the biological characterization of each subtype. Here, we searched for unique genes, motifs, and transcripts in each subtype to investigate the differences of subtypes in each experiment. The 3-nucleotide motifs of each mutation were also constructed for further mutational signature analysis. Furthermore, pathways and GOs of associated genes were analyzed to discover molecular and functional characteristics of each subtype. We also examined molecular data that was available for a subset of the pancreatic cancer samples. Lastly, we investigated the difference between survival curves of PC subtypes.

### 3.4. Motif Rates and Signatures Analysis

There are 96 different types of mutations concerning their 3-nucleotide motifs in DNA. The occurrence rate of these motifs in subtypes is related to specific molecular mechanisms behind mutations in cancers [28]. To study the 3-nucleotide motifs rates in PC subtypes, the relative frequency of each 3-nucleotide motif among samples in each subtype was calculated and plotted in Figure S3. 

**Figure 3:**
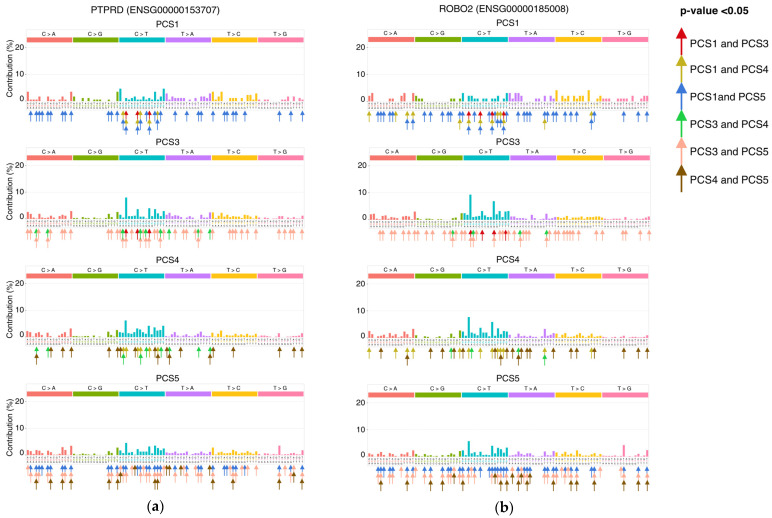
Significantly different motifs in common associated genes. In total, 426 genes are associated with more than two subtypes. However, they have mutated in different motifs. (**a**) PTPRD and (**b**) ROBO2 are two oncogenes that are good examples of this phenomenon. Although they are associated with four subtypes, as it is evident in their bar plot of motif rates, there are multiple differently mutated motifs when rates in subtypes are compared. Each arrow represents a significant difference in the rate of occurrence of the motif that is pointed to, and the color of the arrow indicates the comparison that motif was significant in. The *p*-values can be found in Table S4.

In our gene association analysis, we identified several genes that were significantly mutated in multiple subtypes. Here, we explore if the mutations in these genes have different motif preferences in each subtype. To do this, we investigated the mutational load in 3-nucleotide motifs in the highly mutated genes that were associated to multiple subtypes. As shown in Figure 3, even though some genes are associated with more than one subtype, the mutations within these genes are enriched in different 3-nucleotide motifs. We showed two oncogenes which were associated with more than one subtype in Figure 3. As demonstrated in this figure, *PTPRD* has significantly different motifs when the rates of each motif in subtypes are being considered. CT-A.G, CT-C.G, and CT-G.G occurred more frequently in PCS3, compared to PCS1. In *ROBO2* gene, four motifs (CG-T.A, CT-A.T, TA-A.T, and TA-T.A) occurred more frequently in PCS4, compared to PCS3. We also found 21 different motifs in PCS4 and PCS5. A full list of genes that significantly mutated in multiple subtypes, but in different 3-nucleotide motifs, is provided in Table S4.

**Figure 4:**
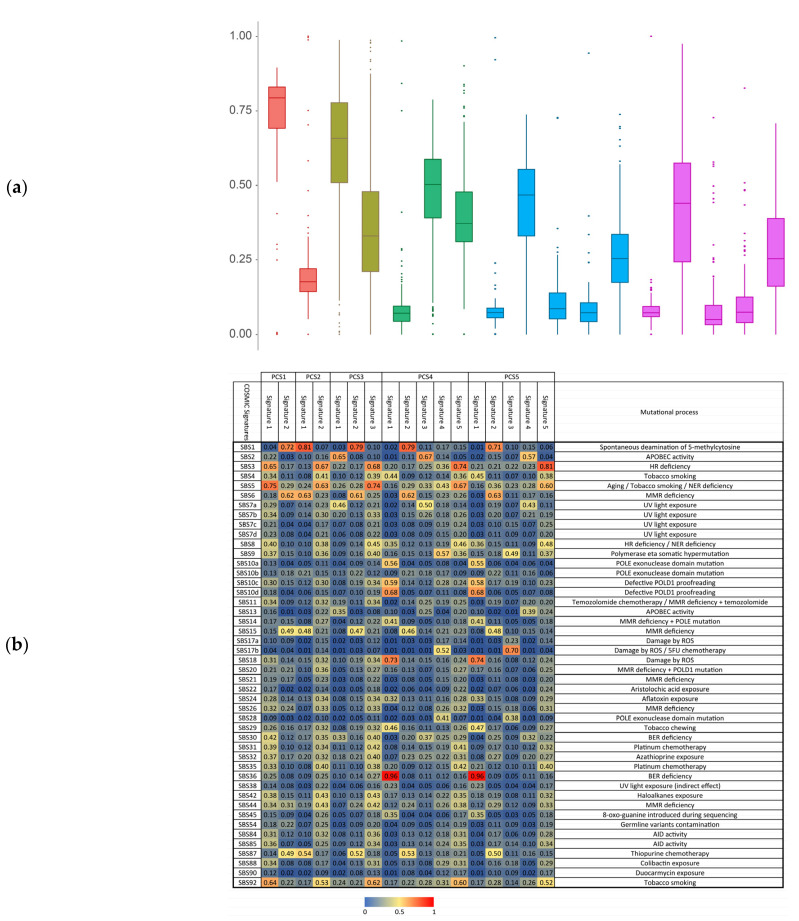
Signature analysis. (**a**) Exposure of samples to signatures. Exposure of each sample to each signature indicates the engagement level of a sample. For example, samples of PCS5 are more exposed to signature 2 of this subtype. This indicates that the molecular mechanism associated with this signature has potentially more affected samples of this subtype. (**b**) Comparing deciphered signatures to COSMIC signatures. This comparison can lead to revealing associated molecular mechanisms causing PC subtype signatures. Each cell of this heatmap indicates a level of similarity.

We also investigated the mutational signatures in each PC subtype, separately [28]. Alexandrov et al. studied mutational signatures to find molecular mechanisms concerning the occurrence of each signature [43]. As it was discussed in [43], different signatures can be interpreted as different molecular mechanisms of mutations. Here, we used CANCERSIGN tool [27] to identify mutational signatures in the identified PC subtypes. Patterns of extracted mutational signatures are provided in Figure S7 (considering Alexnadrov et al. signatures profile, we excluded unknown and artifact signatures from our analysis) [28]. The importance and commonality of signatures in each subtype are shown in terms of boxplots of levels of exposures of samples in Figure 4a. We also calculated the angular similarity between identified signatures in each subtype and the signatures reported by Alexandrov et al. [17,43]. In total, 12 signatures in our study had angular similarity more than 70% with Alexandrov’s *signatures*. SBS1, a spontaneous deamination of 5-methylcytosine was presented in all the subtypes (signature 3 of PCS1 with 72% similarity, signature 1 of PCS2 with 81% similarity, signature 2 of PCS3 with 79% similarity, signature 2 of PCS4 with 87% similarity, and signature 2 of PCS5 with 71% similarity). This signature is potentially associated with the most active mutational molecular mechanism in PC and is related to spontaneous or enzymatic deamination of DNA in which the failure in its detection causes fixation of T substitution for C, before the DNA replication (Figure 4b).

SBS3 is presented in PCS4 and PCS5 (with similarity rate 74% and 81% with PCS4 and PCS5, respectively). This is a defective homologous recombination-based DNA damage repair. SBS3 in pancreatic cancer is related to responders to platinum therapy. Our clinical investigation for these two subtypes revealed that most of the patients in these subtypes were under platinum therapy. Our analysis also showed that SBS5 was presented in PCS1 and PCS3 with similarity rates more than 75% and 74% to PCS1 and PCS3, respectively. This signature is associated to tobacco smoking. Interestingly, we found genes *PDE4D* and HECW1 are the highly mutated genes in PCS1 and PCS3, respectively. Mutations in these genes are known to be associated with smoking behavior [44,45]. SBS17b is only presented in PCS5 (with similarity rate 70%). This signature is possibly associated to fluorouracil (5FU) chemotherapy treatment. Interestingly, we found out that at least 29% of patients in this subtype were under chemotherapy treatment. SBS18 and SBS36 are other Alexandrov’s signatures that are highly associated with subtypes PCS4 and PCS5, suggesting these two subtypes are also under pressure of DNA damage due to reactive oxygen species or somatic *MUTYH* mutations. 

### 3.5. The Mutational Rate in Transcripts

**Figure 5:**
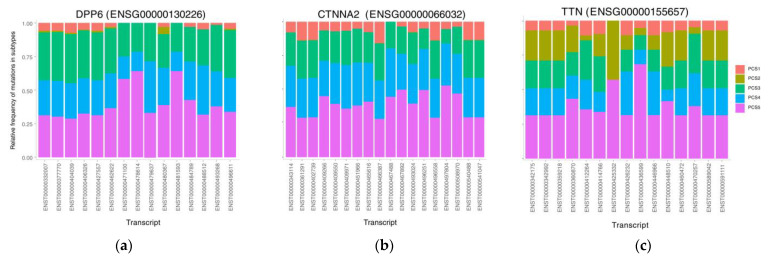
Rate of mutated transcripts in subtypes. Some subtypes tend to mutate more in some transcripts of a gene. This can lead to different outcomes in subtypes. (**a**) gene DPP6. (**b**) gene CTNNA2. (**c**) gene TTN..

Mutations in genes can affect their transcripts and consequently their corresponding proteins based on their respective transcripts. To investigate the effect of mutations concerning transcripts in pancreatic cancer subtypes, we calculated the difference between our identified subtypes concerning the mutational load in different transcripts of the coding genes. Our analyses showed that for many of the candidate protein-coding genes, the mutations occurred in specific transcripts of the genes. To this end, the somatic point mutations were enriched in different transcripts of the genes. For instance, although *CTNNA2* has a 100% mutation rate in both PCS3 and PCS5, their mutational patterns were different from their transcripts (Figure 5). In PCS3, 52% of samples mutated in transcript ENST00000493024, while 88% of samples in PCS5 have mutated in the same transcript. In the *TTN* gene, PCS2 samples had more mutation compared to the *CTNNA2* gene. Interestingly, in various transcripts of this gene, the rates of mutation in subtypes are different. Another interesting point is that in transcript ENST00000425332 only PCS5 and PCS2 had mutations, while the other three subtypes had no mutation at all. In 4 out of 15 transcripts of the *DPP6* gene, only PCS3, PCS4 and PCS5 had the mutation and the other two subtypes had none. This analysis revealed the importance of de novo somatic mutational load in transcripts, which can be used in a better understanding of the underlying mechanisms.

### 3.6. Gene Expression Analysis and Finding Differentially Expressed Genes

**Figure 6:**
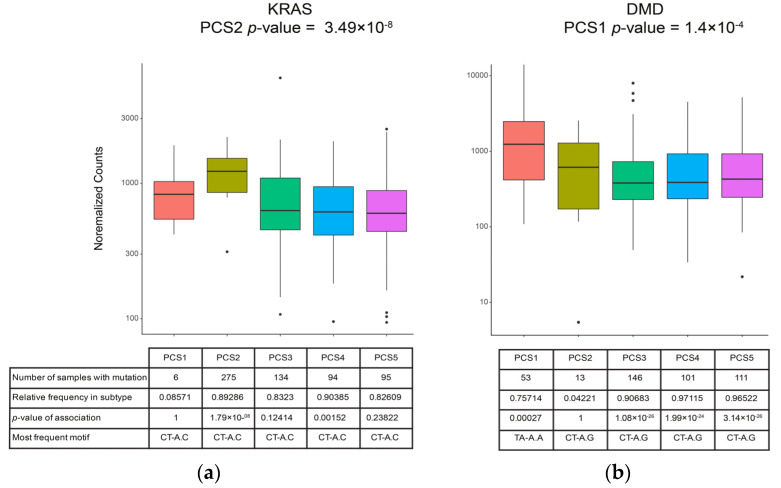
Expression boxplots, mutation, and motif. Expression levels of (**a**) *KRAS* and (**b**) *DMD* in five subtypes are illustrated in the form of boxplots. Some information about mutations in the genome is also provided in tables under each boxplot to represent the potential association of mutation (and their types) on expression levels.

To study the impact of de novo mutations in the PCS subtypes on the expression level of coding genes, RNA-seq data of 307 samples were analyzed. This analysis was conducted by using the DEseq2 package [31] and its defined workflow (*see methods section*). The gene expression level of each gene in PC subtypes was compared to the level of gene expression in other subtypes. As a result, we identified 303 uniquely differentially expressed genes (UDEG) in PCS1, 2427 UDEG genes in PCS2, 267 UDEG genes in PCS3, 136, and 940 UDEG genes in PCS4, and PCS5, respectively. For example, *KRAS* was differentially expressed in PCS2 only; interestingly, it is the only gene that was significantly mutated to this subtype. Another example is *DMD* gene in PCS1. This gene has a tumor suppression activity, and alterations in the expression of this gene in pancreatic tumors have been discussed in [46]. Boxplots of expression of these two genes in the five subtypes are shown in Figure 6. A list of UDEGs for each subtype is provided in Table S13.

### 3.7. Gene Ontology and Pathway Analyses

We then performed gene ontology (GO) and gene pathway analyses to investigate whether candidate genes in PCS subtypes are significantly associated with any specific term [47]. We employed gene set enrichment analysis for all associated genes (see methods section) for each subtype. There are some common GO terms in two or more subtypes and some unique terms for each subtype as listed in Table S14. For example, “regulation of protein kinase C signaling (GO:0090036)” is only associated with PCS3, “DNA damage response, signal transduction by p53 class mediator resulting in cell cycle arrest (GO:0006977)” and “G1 DNA damage checkpoint (GO:0044783)” are unique to PCS4. On the contrary, the “epidermal growth factor receptor signaling pathway (GO:0007173)” is an example of common gene ontologies enriched in subtypes PCS2 and PSC5. Some terms like “regulation of cell proliferation (GO:0042127)” are common in all subtypes. Interestingly, a large number of GO terms across subtypes are related to the nervous system and axons, and there is a known relation between pancreatic nerve alterations and pancreatic cancer. These alterations in size and density of nerves have also some effects on non-neural pancreatic cells [48]. A table of all GO terms related to genes of each subtype is provided in Table S14.

Our pathway analysis also revealed known pathways that are related to pancreatic cancer. For example, the cell adhesion molecules (CAMs) pathway that is enriched in PCS3, PCS4, and PCS5, is a well-known pathway in PC development [49,50,51]. The axon guidance pathway was also enriched in all subtypes. Mutations and other genomic variations were seen in genes of this pathway for PC samples and have been shown to have a critical role in the PC mechanism [52]. Pathways related to associated genes of each subtype are provided in Table S15. We also provided a summary of known cancer related pathways that overlapped with the pathways identified in our study (Figure S8).

### 3.8. Clinical Report and Survival Analysis of the Identified Subtypes

To study and understand the characteristics of each pancreatic cancer subtype, we also examined clinical data and phenotypic information including age, gender, and location of the samples. Moreover, we studied the impact of tumor information including stage and grade on the survival of patients of each subtype, to gain more insight on their effect on patient’s health status. To this end, the frequency of samples for each project in each subtype was counted to investigate if any meaningful relationships between this information and subtypes can be observed. The frequencies of gender and project are provided in Tables S6 and S7 which illustrate distinguishable patterns among our identified subtypes.

As demonstrated in Tables S6 and S7, about 70% of all endocrine samples are in PCS1. Pancreatic cancer endocrine neoplasm is a rare type of pancreatic cancer that occurs in less than 1 per 100,000 persons per year in the general population [53]. Other remaining ductal samples (9 out of 70 samples) in this subtype may have similar molecular functionalities to those endocrine samples. Additionally, men are twice as likely as women in this subtype. For PCS2, all samples (except one sample) are of ductal adenocarcinoma type. Pancreatic ductal adenocarcinoma is the most common and more lethal type of pancreatic cancer that is more resistant to drugs and existing treatments [54]. For this subtype, about 75% of samples were gathered from Australia, meaning that this subtype is more likely to happen in this country. Moreover, PCS2 is more common among men (about 58% are men and 48% are women). The majority of samples in PCS3 have a ductal adenocarcinoma type, and one-tenth of this subtype has an endocrine tumor. It is also observed that PCS4 and PCS5 have approximately the same proportion of ductal and endocrine types. This may indicate that these two subtypes are based on a common functionality between ductal and exocrine types. Moreover, 60% of samples of PCS4 are women, and 66% of samples in PCS5 are men.

**Figure 7:**
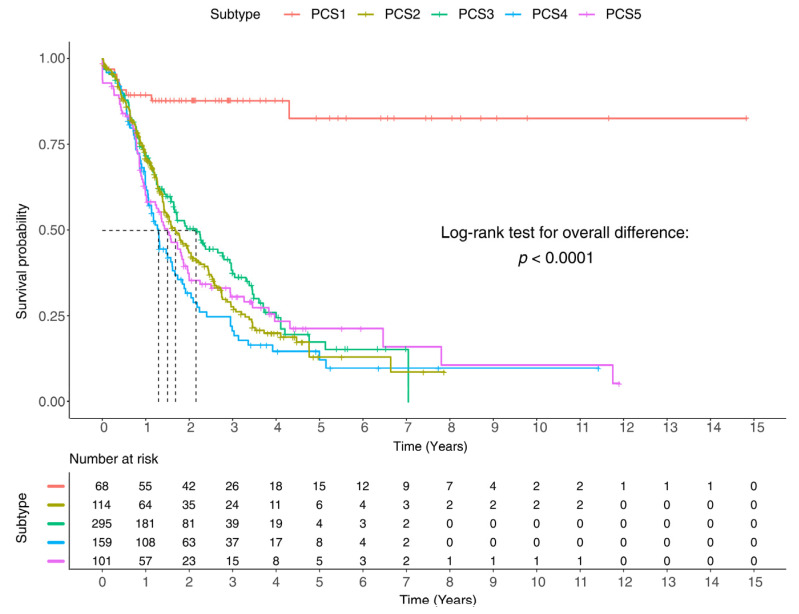
Survival curves. Survival curves of the five subtypes reveal that PCS1 has the longest survival time, and PCS4 has the shortest. For detailed values see Table S8, and for pairwise comparison, survival curves see Table S9.

We next conducted a survival analysis to estimate the overall survival of each subtype. Kaplan–Meier curves of each subtype are shown in Figure 7. The overall survival of PCS1 could not be estimated by its median due to their large amount of censorship. PCS1 mostly contains samples with an endocrine type that is the least lethal type of pancreatic cancer. Hence, donors with this type of cancer may skip follow-ups due to their good health conditions which in turn cause censorship. However, the mean overall survival time of the endocrine is 12.5 years.

Among other subtypes, PCS3 has the longest overall survival time (25.8 months) which is followed by PCS2 (20.2 months), PCS5 (18 months), and PCS4 (15.5 months) (Table S8). The log-rank test also shows that the overall survival of all subtypes is statistically different (*p*-value of 0.0001). We also made pairwise comparisons between subtypes with multiple testing methods (*see methods section*). Among all subtypes, PCS1 has a significantly different survival curve from others, based on all the testing methods. The survival curve of PCS4 is different from PCS1, PCS2, and PCS3 while indicating some similarities to PCS5. PCS2, PCS3, and PCS5 have similar survival curves (Table S9).

Many factors affect the survival of a pancreatic cancer patient. Among them, clinical and histopathological information can help to improve the assessment of a patient’s health condition and survival time. We employed the Cox proportional hazard models to predict the survival time of subtypes by incorporating information such as age, gender, tumor grade, tumor stage, and our subtype indicators [15]. Their power of improving prediction accuracy was also measured by likelihood ratio tests. Here, we first conducted the Cox proportional hazard model with each variable, separately. These single-variable models show the power of improvement of survival prediction only based on the variables in the model (Table S10). Our results demonstrate that subtype indicators are highly significant in predicting survival (*p*-value < 2.2 × 10^−16^). All other variables also performed well in the single variable models (*p*-value < 1.2 × 10^−16^). There is only one category in the stage variable (stage III), and one category in the grade variable (Undifferentiated) that had non-significant effects in their respective models (*p*-value > 0.05).

We then tested the performance of all variables in a complete model (Table S10). The effects of each variable in a full model are tested by removing them one by one from the complete model and comparing the accuracy of the reduced models with the full model. All variables except age (*p*-value = 0.088) are shown to have a significant effect in prediction accuracy of the complete model (*p*-value < 1.2 × 10^−16^) (Table S11). The results demonstrate that all of these properties (e.g., staging, grading, etc.) are important in the survival time prediction.

## 4. Discussion

Despite the large numbers of whole genome and exome sequencing data that are available for cancers, in particular pancreatic cancer, there are still some ambiguities on the vast number of mutations, their types, and causes, leading to significant challenges in identifying mutational subtypes in pancreatic cancer. However, different mutation rates of samples may shed some light on different molecular mechanisms behind mutations among the groups of patients. Mutation is the hallmark of cancer genomes, and many studies have reported cancer subtyping based on the type of frequently mutated driver genes [14,28,55], or the proportion of mutational processes [28], however, none of these existing cancer subtyping methods consider these features simultaneously. In other words, the sequence context of somatic point mutations in driver genes have not been taken into consideration in cancer subtyping and biomarker discovery. Here, we integrated these two features (frequently mutated genes and sequence context of mutated sites) and implemented a bioinformatics pipeline for pancreatic cancer subtyping using 774 pancreatic cancer samples from ICGC consortia. We found 4211 significantly mutated gene-motifs in the pancreatic cancer samples and used them as the features for clustering, resulting in five subtypes among the pancreatic cancer samples (PCS1 to PCS5). PCS1 is potentially the subtype known as *ADEX* that consists of many samples with the endocrine neoplasm type of PC. *ADEX* tumors are shown to be involved in the upregulation of genes that regulate networks involved in *KRAS* activation, exocrine (*NR5A2* and *RBPJL*), and endocrine differentiation (*NEUROD1* and *NKX2-2*) [9]. *PTPRD* (Protein Tyrosine Phosphatase, Receptor Type D) has been mutated in 81.4% of the patients in this subtype (57 samples out of 70). It is shown that the *PTPRD* gene is a tumor suppressor and mutation/downregulation in this gene was observed in multiple cancers including lung and glioblastoma multiforme (a fatal form of brain cancer) [56]. *PTPRD* acts as a regulator for *STAT3*, which is shown to be activated in colon tumors and cell lines. Mutations in PTPRD restrict its ability to regulate *STAT3*, which promotes cancer progression [57]. PCS2 which is the largest subtype with 308 samples, is a *KRAS* addicted subtype as *KRAS* was mutated in more than 89.28% of samples in this subtype. Previous subtyping studies demonstrated that the *RAS* family are highly mutated genes in the lung, colorectal and pancreatic cancers [58]. Moreover, pancreatic ductal adenocarcinoma (PDAC) was reported as the most RAS-addicted among all cancers, which impacts cell differentiation, proliferation, and apoptosis [58]. The clinical trials using small molecule inhibitors targeting *KRAS*, resulted in promising anti-tumor effect for *KRAS*-mediated subtypes in pancreatic cancer [59].

The other three subtypes have a higher rate of mutation compared to PCS1 and PCS2, we therefore can call these three subtypes as hypermutated subtype. PCS3 samples were highly mutated in *SLIT2* and *ROBO1*. Bailey et al. in the previous pancreatic cancer studies demonstrated that *ROBO*/*SLIT* signaling pathways play contradictory and anti-angiogenic roles in tumorigenesis, endometriosis and renal ischemia-reperfusion injury [9,60,61,62,63]. Therefore, the *ROBO*/*SLIT* signaling pathway might be a promising target in pancreatic cancer therapy. *TP53* was the highly mutated gene in PCS4. Previous PC subtyping by Bailey et al. demonstrated that squamous tumors are enriched for *TP53* mutations which interacts with *ASCOM* complex constituents *MLL2* and *MLL3,* and upregulation of the *TP63∆N* transcriptional network [9]. As like as PCS3, PCS5′s samples are also highly mutated in many genes including *ROBO1* and *SLIT2* demonstrating the contribution of *ROBO*/*SLIT* signaling pathway in tumorigenesis of PCS5′s samples. However, mutations in PSC5′s samples were also enriched in *ROBO2*, which is a stroma suppressor gene in the pancreas and acts via TGF-β signaling [64], which may suggest that both *ROBO*/*SLIT* and TGF-β signaling pathways play a role in tumorigenesis of PCS5. A previous study on pancreatitis and PDAC mouse models showed that Robo2 can act as a stroma suppressor gene by restraining myofibroblast activation and T-cell infiltration [64].

Our pathway analysis also revealed cell cycle and the axon guidance pathways as the most common pathways in all PC subtypes identified in this study. Interestingly, the axon guidance pathway was previously observed in murine Sleeping Beauty transposon-mediated somatic mutagenesis models of pancreatic cancer. In addition to common pathways, some subtype-specific pathways were also seen. For example, we identified Protein kinase C signaling pathway, EGFR (epidermal growth factor receptor) signaling pathway and p53 signaling pathway and as potential targets for treatment of the PSC1, PSC2, and PSC4 subtypes, respectively. It is worth mentioning that targeted treatment options are available for some of the pathways observed in our subtypes. For example, cell adhesion molecules (CAMs) are glycoproteins expressed on the surface of cell membranes that act as oncogenes or tumor suppressors in signal transduction; they also act as tumor progression and metastasis regulators [49,50,51]. We identified CAMs as potential therapeutic targets in PCS3, PCS4, and PCS5 subtypes. By considering somatic mutations in all of the genes associated with our PC subtype-specific mutation of signaling pathways, we might be able to find additional cancer patients who could benefit from targeted treatment options. On the one hand, the pathways identified in our analysis are mutated in a large number of PC patients, and on the other hand, targeted treatment options are currently available for most of these pathways. We therefore believe that these treatment options are worthy of further investigation to develop better therapeutic targets.

Our analysis also revealed subtype-specific mutated genes which may be the main cause of functionality among each subtype. Although there are some genes that significantly mutated in multiple subtypes, however, these genes are mutated in different motifs, indicating the context of the mutation is different in these genes in each subtype. This is also true for non-associated genes. For example, about 30% of samples in PCS5 had TTG-to-TGT mutations in *LRP1b* gene while PCS1 and PCS2 had no mutation in this gene-motif, and only 7% of samples in the other two subtypes had mutation in this gene-motif. *PTPRD* is another example that significantly mutated in PCS4 and PCS5 subtypes, however, the mutations were enriched in different motifs in each subtype (Figure 3). This would suggest that, rather than only investigating mutations in well-known oncogenes, we should consider the context of the mutations within driver genes (frequently mutated genes) to accurately identify cancer subtypes as well as targeted treatment biomarkers. By identifying subtype-specific gene-motif profiles, in addition to subtype-specific targeted therapeutics, we may obtain a clearer picture of the molecular mechanisms that cause a high rate of mutations (and consequently a high number of associated genes), in subtypes.

Our mutational signature analysis in the identified subtypes also revealed some new and subtype-specific signatures in addition to the well-known COSMIC signatures in the identified subtypes in this study; these signatures may be utilized to find the molecular mechanisms that are responsible in these subtypes (Figure 4 and Figure S2). These molecular mechanisms systematically make changes across the genome, and hence they can leave a trace of their activity that corresponds to a different rate of motifs. We also found some signatures that are common among all subtypes, but with different exposures. Although the etiology of many COSMIC signatures is still unknown, some of them contain critical information. For instance, signature 1 of PCS4 and PCS5 are similar to SBS10a of COSMIC, and it is known that samples with this signature are hypermutator. The driver of some signatures such as signature 6 of PCS4 (similar to SBS31 of COSMIC) is chemotherapy with platinum drugs, and the driver of those that are similar to SBS3 and SBS6 of COSMIC, are DNA repair mechanism deficiency. The combination of these molecular mechanisms and their effect becomes dominant and drives cancer to different subtypes.

It is now well known that molecular mechanisms underlying the mutational processes can cause mutations across the genome, blindly, because of their shape and structure. However, different rates of gene-motifs may point to different accessibility of molecular mechanisms to genome in different genes. This can possibly be a result of different epigenetic factors in genes. However, this could not be investigated in this study due to lack of epigenetic data but can be a lead for future works.

With genomic medicine emerging as a routine part of the health system, tumor mutational profiling will help to better understand the underlying genetic causes of cancers. The current treatments options are usually based on assessing single gene mutations. Our study and the proposed pipeline to identify PC subtype associated genes and the context of the mutations within these genes (either by identifying gene-motifs or mutation signatures) could help more precise diagnoses by assigning patients to available therapeutic targets or ongoing clinical trials targeting specific mutations, and identifying subtype-specific pathways that might be useful treatment targets for therapeutic intervention.

The gene expression analysis also revealed genes that are differentially expressed in the subtypes. This may be due to the centrality of associated genes or the genes they affect in the pathways or regulatory (co-expression) network. In other words, expression levels of some of our identified subtypes are only driven by mutations, while some others such as PCS2 and PCS5 are only influenced by mutations besides other factors. To verify this claim, we extracted downstream neighbors of associated genes in pathways of each subtype, up to four levels. We discovered that 16, 65, 27, 19, and 166 of UDEGs of PCS1 to PCS5 are among the neighbor genes, respectively (Table S16). Interestingly, “Pathways in cancer” that has been observed for PCS5 contains 30 PCS5 associated genes 14 UDEGs in PCS5 (see the number of associated genes and UDEGs in each pathway in Table S17). The “RAS signaling pathway” in PCS2 has also the largest number of UDEGs (equal to 20). Interestingly, *KRAS* gene was the only associated gene to PCS2 and has probably a strong effect on the expression alteration. 

Our investigations of clinical information, available for a subset of the patients, revealed an association between the survival time of PC patients and histopathological factors such as grading and staging. For example, PCS1 has the longest survival time, and its curve is differentiated compared to the other subtypes (Figure 7). This is because most PCS1 samples were from the endocrine type of PC that has lower lethality. More investigations on the centers that have collected the samples demonstrate that the PCS2 samples mainly came from Australia, and the PCS5 samples from Canada (60%) (Table S6). There is a possibility that some molecular mechanisms associated with the mutational signatures are influenced or driven by ethnicity or geographical variables. There were also some biases towards genders in some subtypes (Table S7), in which 60% of samples in PCS1 are male, and about 60% of samples in PCS4 are female.

## 5. Conclusions

High-throughput sequencing has provided many improvements in finding the key mutations and molecular events by providing a high number of samples. This will lead to accurate classification of patients based on their mutational profiles, and consequently, and better clinical decisions on their treatment. In this manuscript, we provided a list of subtype-specific gene-motifs which can be useful in better understanding the underlying genetic causes of pancreatic cancer, by exploiting the context of the mutations in the driver genes. Considering the genes with significant mutation rates in PC, and the contexts of the mutations in the genes can provide a more effective and personalized treatment for pancreatic cancer. We showed that our proposed pipeline helps discover mutational patterns associated with cancer related pathways, clinical phenotypes, and potential therapeutic target options for cancer-specific subtypes, as well as mutational patterns that are observed across multiple pancreatic cancer types. Our proposed model and its related codes are publicly available online at: https://github.com/bcb-sut/Pancreatic-Cancer-Subtype-Identification (accessed on 10 August 2021).

## Data Availability

The source code and a sample dataset are available as a supplementary file.

## Supplementary Materials

The following are available online at https://www.mdpi.com/article/10.3390/cancers13174376/s1, Figure S1: Scatterplot of samples in first two Principal Component dimensions, Figure S2: Evaluation plots, Figure S3: Motif rate—main subtypes, Figure S4: Motif rate—outlier subtypes, Figure S5: Venn diagram of associated genes, Figure S6: Venn diagram of DEGs, Figure S7: Signatures of pancreatic cancer subtypes, Figure S8: A summary of known cancer related pathways that overlapped with cancer pathways identified in this study, Table S1: Significant genes, Table S2: Significant gene-motifs, Table S3. Significant features, Table S4: Significantly different motifs in common associated genes, Table S5: All final associated genes, Table S6: Project frequencies, Table S7: Gender frequency, Table S8: Overall survival time estimation, Table S9: Survival pairwise comparison, Table S10: Survival cox regression, Table S11: Survival cox regression likelihood ratio test, Table S12: t-test results, Table S13: Unique differentially expressed genes, Table S14: Gene ontologies (GO), Table S15: Pathways analysis, Table S16: Number of UDEGs among up to fourth-order downstream neighbors of associated genes in pathways, Table S17: The number of associated genes and UDEGs in their neighborhood in pathways.

## References

[1] Siegel R.L., Miller K.D., Jemal A. (2019). Cancer statistics, 2019. CA Cancer J. Clin..

[2] Rahib L., Smith B.D., Aizenberg R., Rosenzweig A.B., Fleshman J.M., Matrisian L.M. (2014). Projecting Cancer Incidence and Deaths to 2030: The Unexpected Burden of Thyroid, Liver, and Pancreas Cancers in the United States. Cancer Res..

[3] Collisson E.A., Bailey P., Chang D.K., Biankin A.V. (2019). Molecular subtypes of pancreatic cancer. Nat. Rev. Gastroenterol. Hepatol..

[4] Slamon D.J., Leyland-Jones B., Shak S., Fuchs H., Paton V., Bajamonde A., Fleming T., Eiermann W., Wolter J., Pegram M. (2001). Use of Chemotherapy plus a Monoclonal Antibody against HER2 for Metastatic Breast Cancer That Overexpresses HER2. N. Engl. J. Med..

[5] Lynch T.J., Bell D.W., Sordella R., Gurubhagavatula S., Okimoto R.A., Brannigan B.W., Harris P.L., Haserlat S.M., Supko J.G., Haluska F.G. (2004). Activating mutations in the epidermal growth factor receptor underlying responsiveness of non-small-cell lung cancer to gefitinib. N. Engl. J. Med..

[6] Garcea G., Neal C., Pattenden C., Steward W., Berry D. (2005). Molecular prognostic markers in pancreatic cancer: A systematic review. Eur. J. Cancer.

[7] Collisson E.A., Sadanandam A., Olson P., Gibb W.J., Truitt M., Gu S., Cooc J., Weinkle J., Kim G.E., Jakkula L. (2011). Subtypes of pancreatic ductal adenocarcinoma and their differing responses to therapy. Nat. Med..

[8] Moffitt R., Marayati R., Flate E.L., Volmar K.E., Loeza S.G.H., Hoadley K., Rashid N.U., Williams L.A., Eaton S.C., Chung A.H. (2015). Virtual microdissection identifies distinct tumor- and stroma-specific subtypes of pancreatic ductal adenocarcinoma. Nat. Genet..

[9] Bailey P., Initiative A.P.C.G., Chang D.K., Nones K., Johns A.L., Patch A.-M., Gingras M.-C., Miller D.K., Christ A.N., Bruxner T.J.C. (2016). Genomic analyses identify molecular subtypes of pancreatic cancer. Nat. Cell Biol..

[10] Sivakumar S., de Santiago I., Chlon L., Markowetz F. (2017). Master Regulators of Oncogenic KRAS Response in Pancreatic Cancer: An Integrative Network Biology Analysis. PLoS Med..

[11] Puleo F., Nicolle R., Blum Y., Cros J., Marisa L., Demetter P., Quertinmont E., Svrcek M., Elarouci N., Iovanna J. (2018). Stratification of Pancreatic Ductal Adenocarcinomas Based on Tumor and Microenvironment Features. Gastroenterology.

[12] Androulakis I.P., Yang E., Almon R.R. (2007). Analysis of Time-Series Gene Expression Data: Methods, Challenges, and Opportunities. Annu. Rev. Biomed. Eng..

[13] Tate J.G., Bamford S., Jubb H.C., Sondka Z., Beare D.M., Bindal N., Boutselakis H., Cole C.G., Creatore C., Dawson E. (2018). COSMIC: The Catalogue Of Somatic Mutations In Cancer. Nucleic Acids Res..

[14] Kuijjer M.L., Paulson J.N., Salzman P., Ding W., Quackenbush J. (2018). Cancer subtype identification using somatic mutation data. Br. J. Cancer.

[15] Kuipers J., Thurnherr T., Moffa G., Suter P., Behr J., Goosen R., Christofori G., Beerenwinkel N. (2018). Mutational interactions define novel cancer subgroups. Nat. Commun..

[16] Waddell N., Pajic M., Patch A.M., Chang D.K., Kassahn K.S., Bailey P., Johns A.L., Miller D., Nones K., Quek K. (2015). Whole genomes redefine the mutational landscape of pancreatic cancer. Nature..

[17] Alexandrov L.B., Kim J., Haradhvala N.J., Huang M.N., Ng A.W.T., Wu Y., Boot A., Covington K.R., Gordenin D.A., Bergstrom E.N. (2020). The repertoire of mutational signatures in human cancer. Nat. Cell Biol..

[18] Parvin H., Minaei B., Alizadeh H., Beigi A. (2011). A Novel Classifier Ensemble Method Based on Class Weightening in Huge Dataset. International Symposium on Neural Networks.

[19] All Codes Related to This Research. https://github.com/bcb-sut/Pancreatic-Cancer-Subtype-Identification.

[20] International Cancer Genome Consortium Data Portal. https://dcc.icgc.org/.

[21] Lawrence M., Huber W., Pagès H., Aboyoun P., Carlson M., Gentleman R., Morgan M., Carey V.J. (2013). Software for Computing and Annotating Genomic Ranges. PLoS Comput. Biol..

[22] Gehring J.S., Fischer B., Lawrence M.F., Huber W. (2015). SomaticSignatures: Inferring mutational signatures from single-nucleotide variants: Figure 1. Bioinformatics.

[23] Dashti H., Dehzangi A., Bayati M., Breen J., Lovell N., Ebrahimi D., Rabiee R.H., Alinejad-Rokny H. (2020). Integrative analysis of mutated genes and mutational processes reveals seven colorectal cancer subtypes. bioRxiv.

[24] Scrucca L., Fop M., Murphy T.B., Adrian E.R. (2016). mclust 5: Clustering, Classification and Density Estimation Using Gaussian Finite Mixture Models. R J..

[25] Fraley C., Raftery A.E. (2007). Model-based methods of classification: Using the mclust software in chemometrics. J. Stat. Softw..

[26] Fraley C., Raftery A.E. (1998). How Many Clusters? Which Clustering Method? Answers Via Model-Based Cluster Analysis. Comput. J..

[27] Bayati M., Rabiee H.R., Mehrbod M., Vafaee F., Ebrahimi D., Forrest A., Alinejad-Rokny H. (2020). CANCERSIGN: A user-friendly and robust tool for identification and classification of mutational signatures and patterns in cancer genomes. Sci. Rep..

[28] Alexandrov L.B., Nik-Zainal S., Wedge D.C., Aparicio S.A., Behjati S., Biankin A.V., Bignell G.R., Bolli N., Borg A., Børresen-Dale A.L. (2013). Signatures of mutational processes in human cancer. Nature.

[29] Shamshirband S., Fathi M., Dehzangi A., Chronopoulose A.T. (2020). A review on deep learning approaches in healthcare systems: Taxonomies, challenges, and open issues. J. Biomed. Inf..

[30] Ebrahimi D., Alinejad-Rokny H., Davenport M.P. (2014). Insights into the Motif Preference of APOBEC3 Enzymes. PLoS ONE.

[31] Love M.I., Huber W., Anders S. (2014). Moderated estimation of fold change and dispersion for RNA-seq data with DESeq2. Genome Biol..

[32] Enrichr. https://amp.pharm.mssm.edu/Enrichr/.

[33] Javanmard R., Jeddisaravi K., Alinejad-Rokny H. (2013). Proposed a New Method for Rules Extraction Using Artificial Neural Network and Artificial Immune System in Cancer Diagnosis. J. Bionanoscience.

[34] Rad M.P., Pourshaikh R. (2012). Conceptual Information Retrieval in Cross-Language Searches. Res. J. Appl. Sci. Eng. Technol..

[35] Alinejad-Rokny H., Parvin S. (2011). Divide and Conquer Classification. Int. J. Sci. Basic Appl. Res..

[36] Hasanzadeh E., Rokny H.A., Poyan M. (2012). Text clustering on latent semantic indexing with particle swarm optimization (PSO) algorithm. Int. J. Phys. Sci..

[37] Esmaeili L., Minaei-Bidgoli B., Alinejad-Rokny H., Nasiri M. (2012). Hybrid recommender system for joining virtual communities. Res. J. Appl. Sci. Eng. Technol..

[38] Alinejad-Rokny H., Anwar F., Waters S., Davenport M., Ebrahimi D. (2016). Source of CpG Depletion in the HIV-1 Genome. Mol. Biol. Evol..

[39] Parvin H., MirnabiBaboli M., Alinejad-Rokny H. (2015). Proposing a classifier ensemble framework based on classifier selection and decision tree. Eng. Appl. Artif. Intell..

[40] Woolson R.F. (1981). Rank Tests and a One-Sample Logrank Test for Comparing Observed Survival Data to a Standard Population. Biometrics.

[41] Karadeniz P.G., Ercan I. (2017). Examining Tests for Comparing Survival Curves with Right Censored Data. Stat. Transit. New Ser..

[42] Zhu Y., Neeman T., Yap V.B., Huttley G.A. (2017). Statistical Methods for Identifying Sequence Motifs Affecting Point Mutations. Genetics.

[43] Alexandrov L.B., Kim J., Haradhvala N.J., Huang M.N., Ng A.W., Wu Y., Boot A., Covington K.R., Gordenin D.A., Bergstrom E.N. (2019). The repertoire of mutational signatures in human cancer. BioRxiv.

[44] Zuo H., Han B., Poppinga W.J., Ringnalda L., Kistemaker L.E.M., Halayko A.J., Gosens R., Nikolaev O.V., Schmidt M. (2017). Cigarette smoke up-regulates PDE3 and PDE4 to decrease cAMP in airway cells. Br. J. Pharmacol..

[45] Park S.L., Carmella S.G., Chen M., Patel Y., Stram D.O., Haiman C.A., Le Marchand L., Hecht S.S. (2015). Mercapturic Acids Derived from the Toxicants Acrolein and Crotonaldehyde in the Urine of Cigarette Smokers from Five Ethnic Groups with Differing Risks for Lung Cancer. PLoS ONE.

[46] Luce L.N., Abbate M., Cotignola J., Giliberto F. (2016). Non-myogenic tumors display altered expression of dystrophin (DMD) and a high frequency of genetic alterations. Oncotarget.

[47] (2016). The Gene Ontology Consortium, Expansion of the Gene Ontology knowledgebase and resources. Nucleic Acids Res..

[48] Demir I.E., Friess H., Ceyhan G.O. (2015). Neural plasticity in pancreatitis and pancreatic cancer. Nat. Rev. Gastroenterol. Hepatol..

[49] Moh M.C., Shen S. (2009). The roles of cell adhesion molecules in tumor suppression and cell migration: A new paradox. Cell Adhes. Migr..

[50] Bassagañas S., Carvalho S., Dias A., Pérez-Garay M., Ortiz R., Figueras J., Reis C., Pinho S.S., Peracaula R. (2014). Pancreatic Cancer Cell Glycosylation Regulates Cell Adhesion and Invasion through the Modulation of α2β1 Integrin and E-Cadherin Function. PLoS ONE.

[51] Farahani E., Patra H.K., Jangamreddy J.R., Rashedi I., Kawalec M., Pariti R.K.R., Batakis P., Wiechec E. (2014). Cell adhesion molecules and their relation to (cancer) cell stemness. Carcinog..

[52] Biankin A.V., Initiative A.P.C.G., Waddell N., Kassahn K.S., Gingras M.-C., Muthuswamy L.B., Johns A.L., Miller D.K., Wilson P.J., Patch A.-M. (2012). Pancreatic cancer genomes reveal aberrations in axon guidance pathway genes. Nat. Cell Biol..

[53] Halfdanarson T.R., Rubin J., Farnell M.B., Grant C.S., Petersen G.M. (2008). Pancreatic endocrine neoplasms: Epidemiology and prognosis of pancreatic endocrine tumors. Endocr.-Relat. Cancer.

[54] Ilic M., Ilic I. (2016). Epidemiology of pancreatic cancer. World J. Gastroenterol..

[55] Dietlein F., Weghorn D., Taylor-Weiner A., Richters A., Reardon B., Liu D., Lander E.S., Van Allen E.M., Sunyaev S.R. (2020). Identification of cancer driver genes based on nucleotide context. Nat. Genet..

[56] Veeriah S., Brennan C., Meng S., Singh B., Fagin J.A., Solit D.B., Paty P.B., Rohle D., Vivanco I., Chmielecki J. (2009). The tyrosine phosphatase PTPRD is a tumor suppressor that is frequently inactivated and mutated in glioblastoma and other human cancers. Proc. Natl. Acad. Sci. USA.

[57] Funato K., Yamazumi Y., Oda T., Akiyama T. (2011). Tyrosine phosphatase PTPRD suppresses colon cancer cell migration in coordination with CD44. Exp. Ther. Med..

[58] Waters A.M., Der C.J. (2017). KRAS: The Critical Driver and Therapeutic Target for Pancreatic cancer. Cold Spring Harb. Perspect. Med..

[59] Canon J., Rex K., Saiki A., Mohr C., Cooke K., Bagal D., Gaida K., Holt T., Knutson C.G., Koppada N. (2019). The clinical KRAS(G12C) inhibitor AMG 510 drives anti-tumour immunity. Nat. Cell Biol..

[60] Guo S.-W., Zheng Y., Lu Y., Liu X., Geng J.-G. (2013). Slit2 overexpression results in increased microvessel density and lesion size in mice with induced endometriosis. Reprod. Sci..

[61] Rama N., Dubrac A., Mathivet T., Chárthaigh R.-A.N., Genet G., Cristofaro B., Pibouin-Fragner L., Ma L., Eichmann A., Chédotal A. (2015). Slit2 signaling through Robo1 and Robo2 is required for retinal neovascularization. Nat. Med..

[62] Li S., Huang L., Sun Y., Bai Y., Yang F., Yu W., Li F., Zhang Q., Wang B., Geng J. (2015). Slit2 Promotes Angiogenic Activity Via the Robo1-VEGFR2-ERK1/2 Pathway in Both In Vivo and In Vitro Studies. Investig. Ophthalmol. Vis. Sci..

[63] Chaturvedi S., Yuen D.A., Bajwa A., Huang Y.-W., Sokollik C., Huang L., Lam G.Y., Tole S., Liu G.-Y., Pan J. (2013). Slit2 Prevents Neutrophil Recruitment and Renal Ischemia-Reperfusion Injury. J. Am. Soc. Nephrol..

[64] Pinho A.V., Van Bulck M., Chantrill L., Arshi M., Sklyarova T., Herrmann D., Vennin C., Gallego-Ortega D., Mawson A., Giry-Laterriere M. (2018). ROBO2 is a stroma suppressor gene in the pancreas and acts via TGF-β signalling. Nat. Commun..

