# Supplementary material for: Whole-Genome Analysis of De Novo Somatic Point Mutations Reveals Novel Mutational Biomarkers in Pancreatic Cancer
Source: Cancers (Basel). 2021 Aug 30;13(17):4376. doi: 10.3390/cancers13174376 (PMC8431675; doi:10.3390/cancers13174376)
Supplement: Supplementary file 1 [file cancers-13-04376-s001.zip › cancers-1339999-supplementary.pdf]

Supplementary Materials: Whole-Genome Analysis of De Novo Somatic Point Mutations Reveals Novel Mutational Biomarkers in Pancreatic Cancer

Amin Ghareyazi, Amir Mohseni, Hamed Dashti, Abdollah Dehzangi, Amin Beheshti, Hamid R. Rabiee and Hamid Alinejad-Rokny

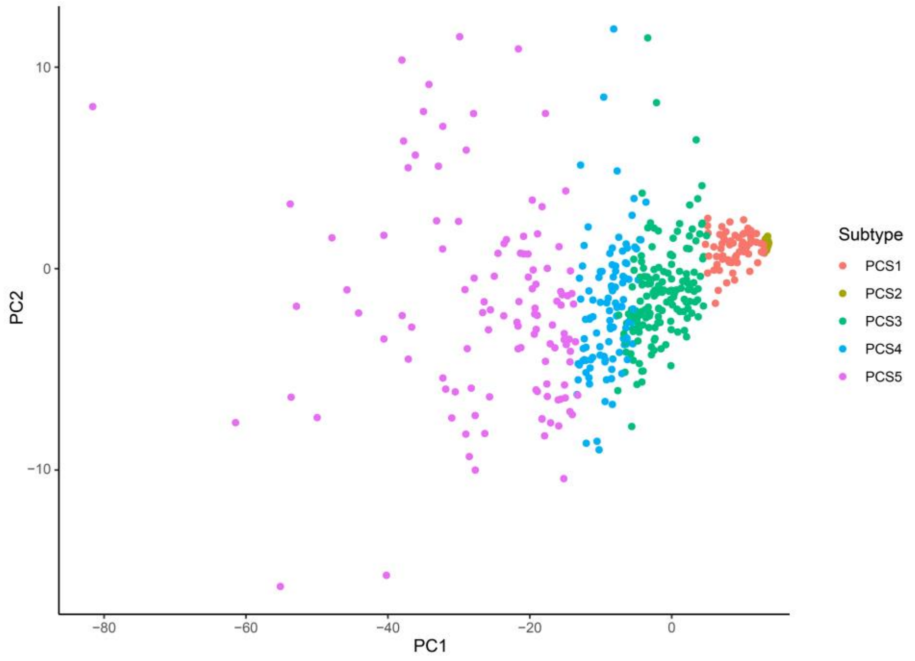

Figure S1. Scatterplot of samples in first two Principal Component dimensions.

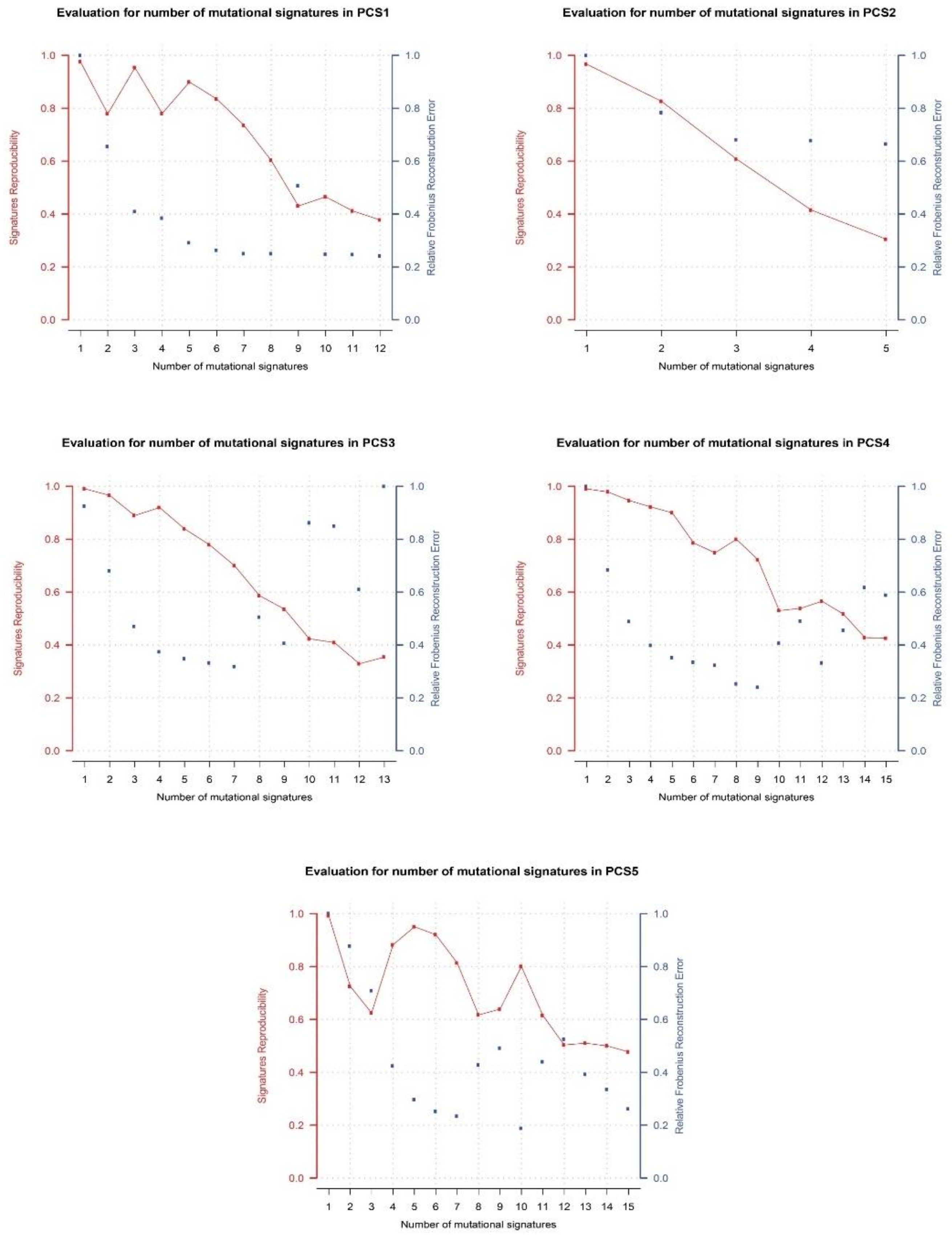

Figure S2. Evaluation plots. Evaluation plots for deciding the number of mutational signatures.

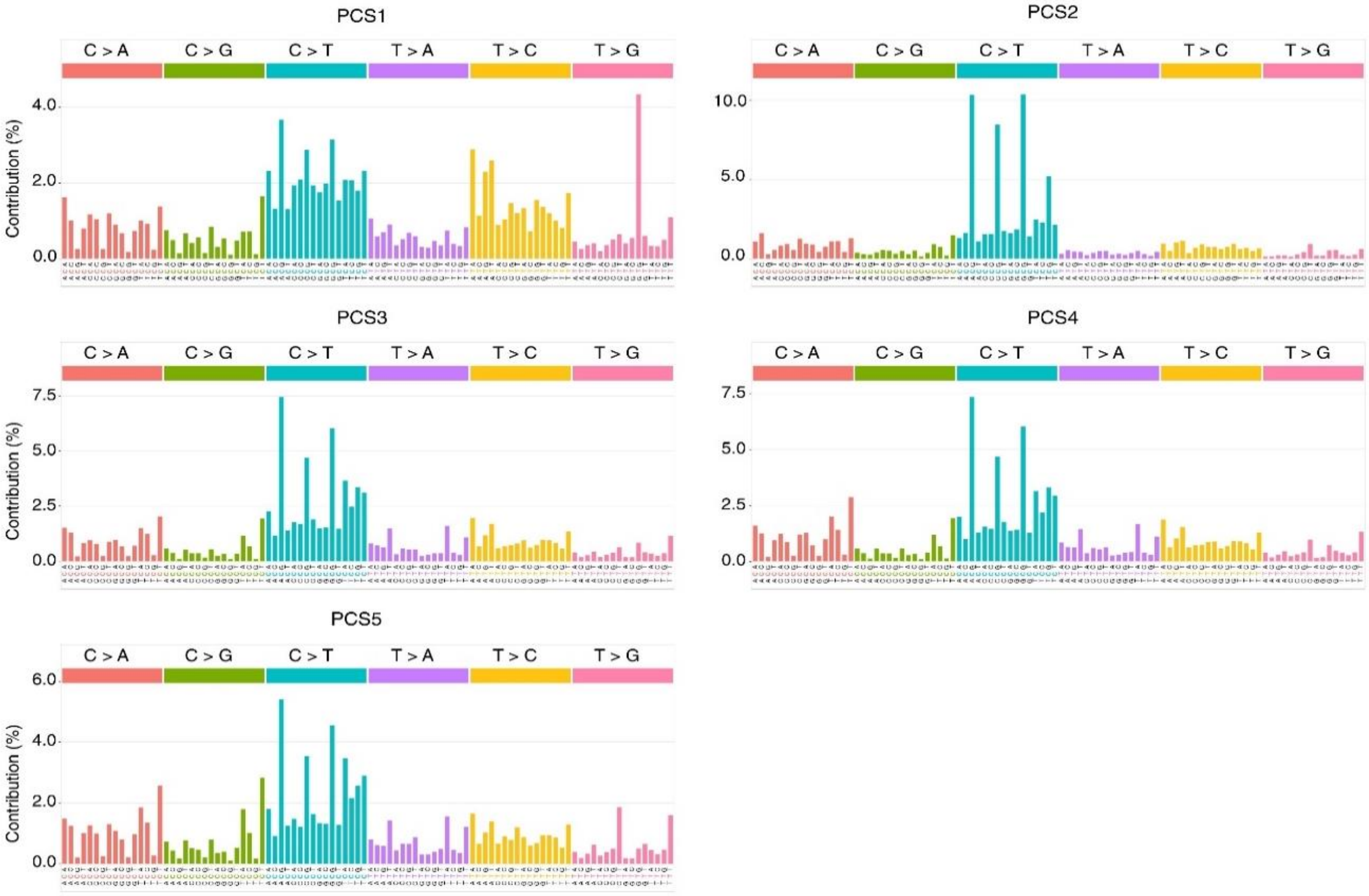

Figure S3. Motif rate—main subtypes. Rate of occurrence of 96 types of 3-mer motifs in 5 subtypes of PC.

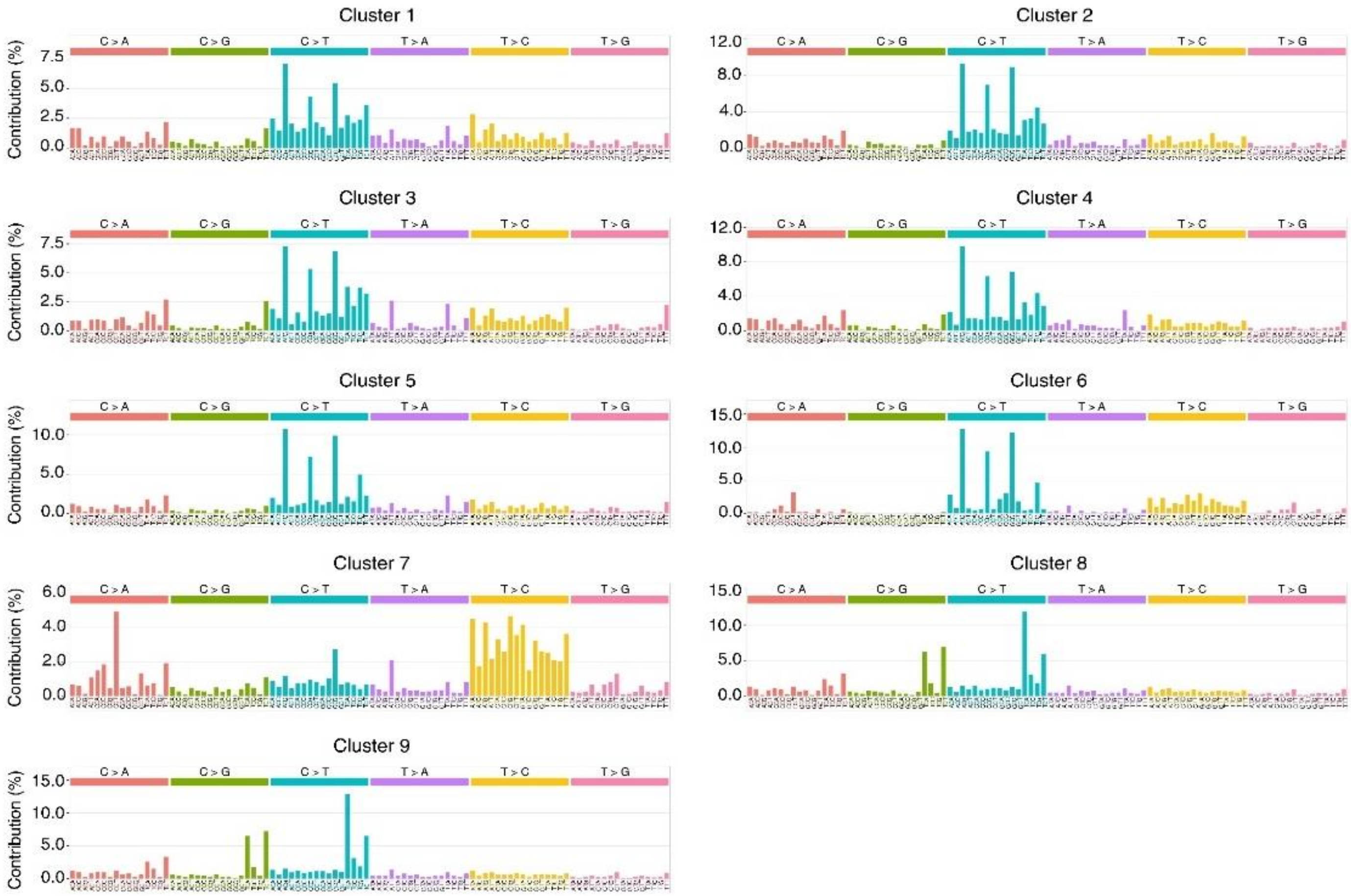

Figure S4. Motif rate—outlier subtypes. Rate of occurrence of 96 types of 3-mer motifs in 9 outlier clusters.

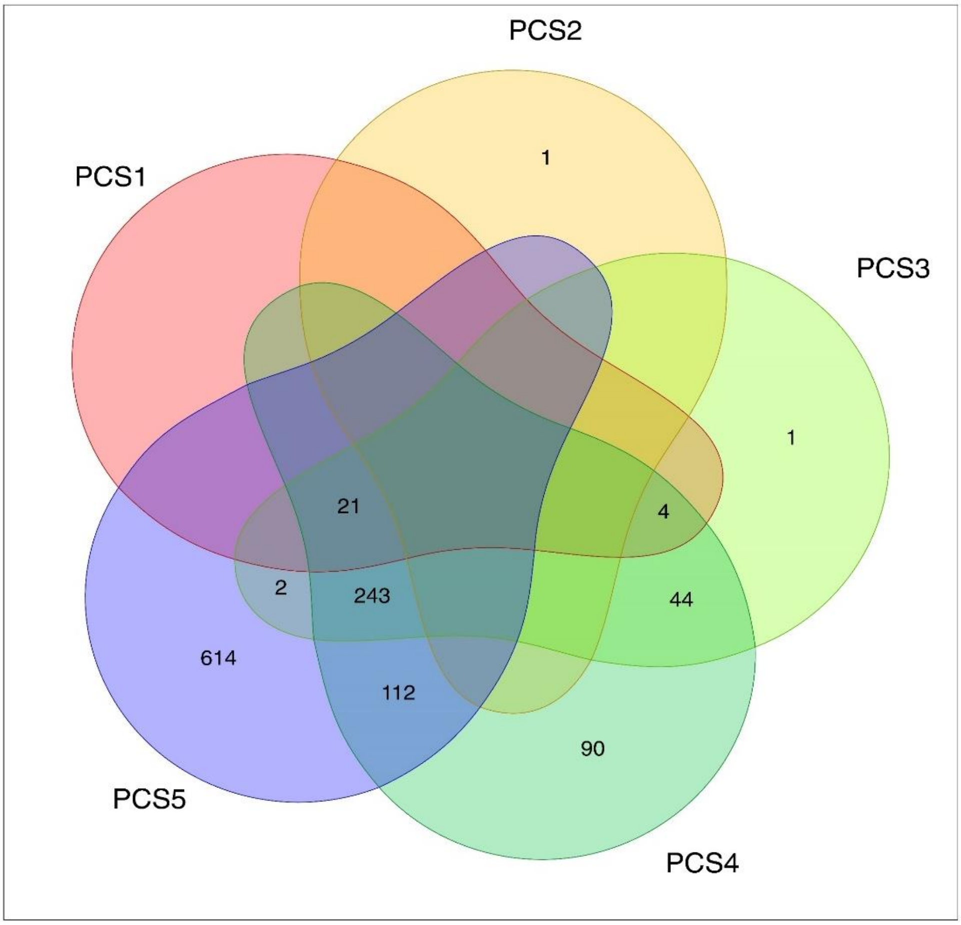

Figure S5. Venn diagram of associated genes. Venn diagram of associated genes of PC subtypes (For more information on gene association study see the Methods section).

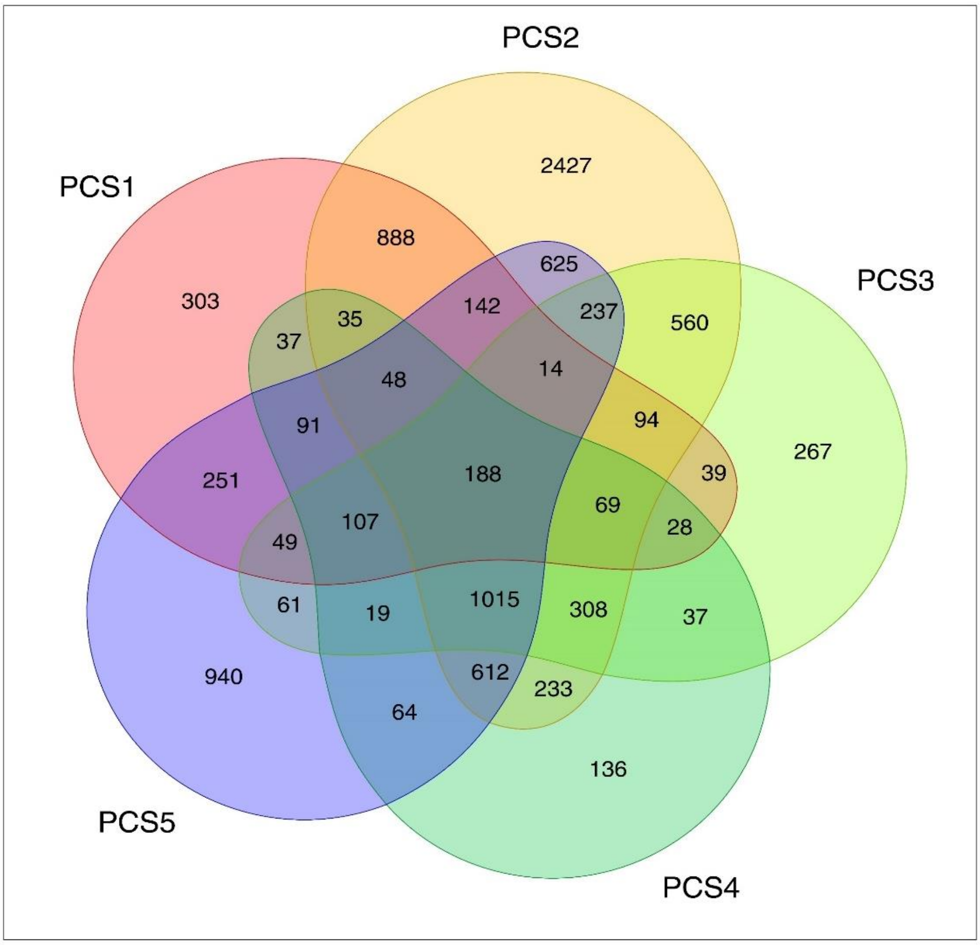

Figure S6. Venn diagram of DEGs. Venn diagram of differentially expressed genes of each subtype. Expression levels of each subtype are compared to all other four subtypes, and DEGs are inferred. Genes that are only in one subtype are considered as UDEGs (uniquely Differentially Expressed Genes) of that subtype.

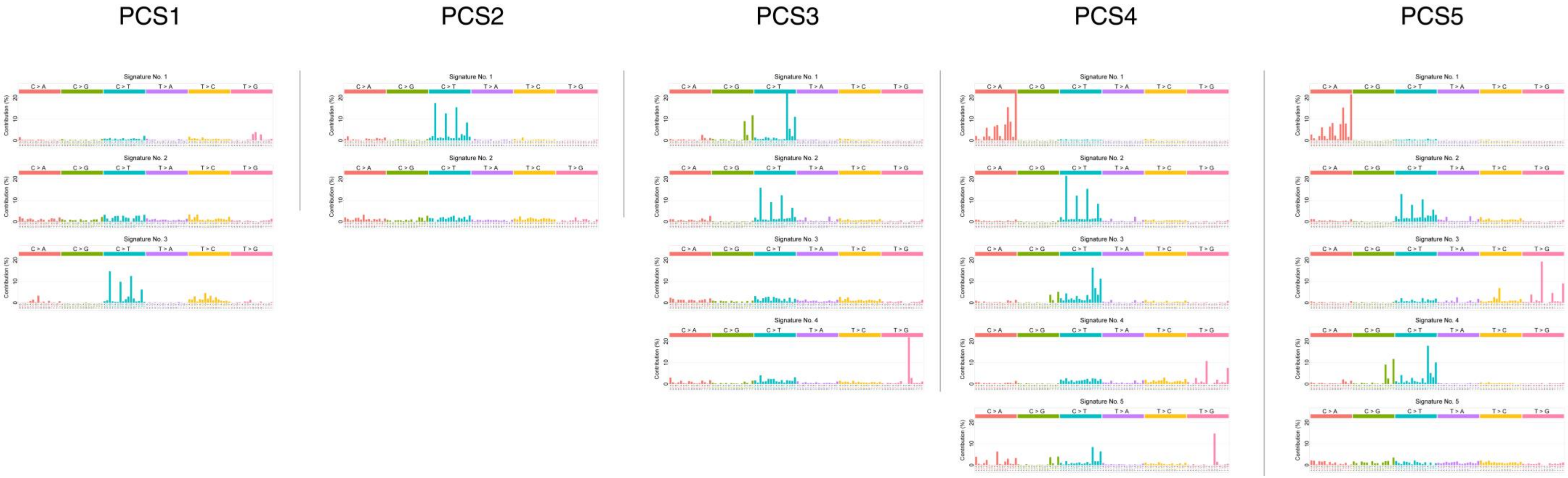

Figure S7. Signatures of pancreatic cancer subtypes. Signatures of PC subtypes extracted by the CANCERSIGN tool. These are prevalent patterns of 3-mer motif of mutations among samples of each subtype.

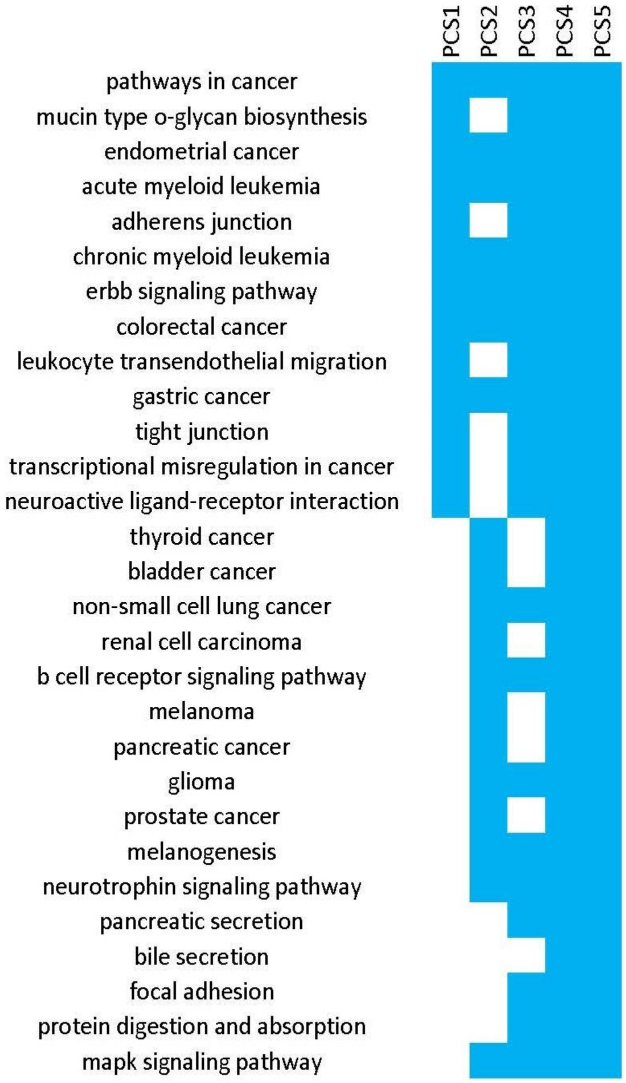

Figure S8. A summary of known cancer related pathways that overlapped with cancer pathways identified in this study.

Table S1. Significant genes.

Table S2. Significant gene-motifs.

Table S3. Significant features.

Table S4. Significantly different motifs in common associated genes.

Table S5. All final associated genes.

Table S6. Project frequencies.

Table S7. Gender frequency.

Table S8. Overall survival time estimation.

Table S9. Survival pairwise comparison.

Table S10. Survival cox regression.

Table S11. Survival cox regression likelihood ratio test.

Table S12. *t*-test results.

Table S13. Unique differentially expressed genes.

Table S14. Gene ontologies (GO).

Table S15. pathways analysis.

Table S16. Number of UDEGs among up to fourth-order downstream neighbors of associated genes in pathways.

Table S17. The number of associated genes and UDEGs in their neighborhood in pathways.

Table S1–S17. are provided separately, attached as an Excel file.
